# Association of antioxidant-added highly cross-linked polyethylene on revision risk: a registry-based study of 198,073 total hip replacements from the Australian Orthopaedic Association National Joint Replacement Registry between 2014 and 2023

**DOI:** 10.2340/17453674.2025.45181

**Published:** 2026-01-23

**Authors:** Peter L LEWIS, David G CAMPBELL, Peiyao DU, Helena OAKEY, Richard N de STEIGER, Paul N SMITH

**Affiliations:** 1 Wakefield Orthopaedic Clinic, University of Adelaide, Adelaide; 2 Australian Orthopaedic Association National Joint Replacement Registry (AOANJRR), Adelaide; 3 South Australian Health and Medical Research Institute (SAHMRI), Adelaide;; 4 The University of Melbourne, Epworth Hospital, Melbourne, Victoria; 5 Australian National University, Canberra, Australia

## Abstract

**Background and purpose:**

Adding antioxidant to highly cross-linked polyethylene (XLPE) is proposed to improve oxidation resistance and decrease wear in total hip replacements (THR), but long-term performance is unknown. We aimed to compare the revision rates of THR using cementless acetabular components where the insert was made of either XLPE with antioxidant (AOXLPE) or XLPE, using data from a large national registry.

**Methods:**

The population was THR from the Australian Orthopaedic Association National Joint Replacement Registry (AOANJRR) in the 10-year period 2014–2023 with modular cementless acetabular components and ceramic or metal femoral heads used for osteoarthritis. We compared primary THR using XLPE with antioxidant (AOXLPE) acetabular inserts with XLPE acetabular inserts. The outcome measured was all-cause revision. Cumulative percentage revision (CPR) was calculated using the Kaplan–Meier method, and comparisons made using Cox proportional hazards models.

**Results:**

There were 198,073 THRs, of which 35,309 had AOXLPE inserts. There were 769 and 4,327 revisions with AOXLPE and XLPE inserts, respectively. While there was no early difference, the AOXLPE group had a lower revision rate after 3 years (HR 0.64, 95% confidence interval [CI] 0.48–0.84). When adjusted for multiple factors the AOXLPE group still had a lower revision rate after 3 years (HR 0.63, CI 0.47–0.83). Revisions for loosening, wear-related causes, and fracture were proportionately lower in the AOXLPE group, but no difference was found with revisions for dislocation/instability or infection.

**Conclusion:**

While there was no early difference, THR with AOXLPE acetabular inserts had a lower revision rate after 3 years than XLPE. This suggests a possible clinical benefit using AOXLPE but the difference may, in part, be related to the associated femoral or acetabular components.

Total hip replacement (THR) is a successful treatment for severe osteoarthritis [1], and survivorship has improved since highly cross-linked polyethylene (XLPE) was introduced [2,3]. However, cross-linking by irradiation creates free radicals that can remain in the material [4] and cause oxidative degeneration of polyethylene [5]. Thermal treatments to improve oxidative stability lower mechanical strength [4]. Adding antioxidant to polyethylene (AOXLPE) may mitigate oxidation without lowering the mechanical properties [4], and also decrease oxidation-induced inflammatory response in the periprosthetic tissues [6]. Despite the theoretical advantages of AOXLPE, the clinical benefit is unclear.

Radiographic studies comparing XLPE with and without antioxidant have shown lower femoral head penetration measured at 5–7 years, but no difference has been found for revision rates or patient outcome scores [7-9]. Meta-analyses have revealed similar femoral head penetration findings when comparing AOXLPE with either “standard” polyethylene (ultra-high molecular weight polyethylene-UHMWPE) or XLPE, but all-cause revision has less frequently been studied and the results have been mixed [10-12]. It is suggested that larger studies with longer follow-up are required to show potential differences [5,12].

The aim of this study was to compare the revision rates of THR using cementless acetabular components where the insert was made of either AOXLPE or XLPE, using data from a large national registry.

## Methods

### Study design

We used data from the Australian Orthopaedic Association National Joint Replacement Registry (AOANJRR). The AOANJRR commenced data collection on September 1, 1999, achieving complete national implementation by mid-2002. The registry obtains an almost complete dataset (98.8%) of hip, knee, and shoulder replacement performed in Australia [13]. AOANJRR data is externally validated against patient-level data provided by all Australian state and territory health departments. Data is also matched bi-annually with the Australian Government’s National Death Index (NDI) to obtain information on the date of death. The study was conducted using the STROBE (Strengthening Reporting of Observational Studies in Epidemiology) guidelines for observational studies.

### Participants

The population for this study consisted of all primary THR procedures over the 10-year period from 1 January 2014 until the end of December 2023. All-polyethylene cups and non-modular components were excluded. We included THRs with the initial diagnosis of osteoarthritis, with cementless acetabular shells, mixed ceramic or metal femoral heads, and only “modern” components, defined as those that were used in Australia in 2023. Dual mobility and constrained inserts were excluded.

### Exposure

**Figure 1:**
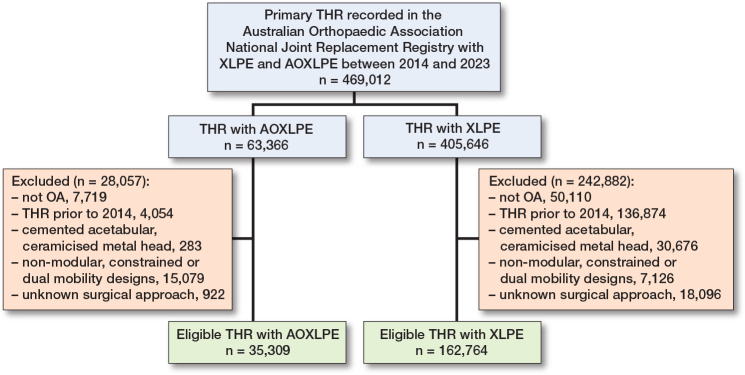
Patient flow diagram.

The exposure of interest was the material of the acetabular insert. Acetabular inserts made of AOXLPE were compared with inserts made of XLPE. AOXLPE included XLPE with antioxidant (commonly Vitamin E) added either by blending or diffusion. XLPE was defined as UHMWPE irradiated by high dose (≥50 kGy) gamma or electron beam radiation. Figure 1 displays a flow diagram showing inclusions/exclusions.

### Outcome

The outcome measured was the cumulative percentage revision (CPR) for any reason. CPR was calculated for THR using AOXLPE or XLPE by the Kaplan–Meier method. Cox proportional hazards models were used to compare revision rates, with the assumption of proportional hazards checked by testing for a significant interaction between each covariate and the log of time. Rates and ratios are reported with 95% confidence intervals (CI). The 2 groups were additionally compared adjusting for age, sex, head size, head material, femoral fixation and surgical approach.

### Statistics

The registry achieves an almost complete capture of hip, knee, and shoulder replacement procedures in Australia—98.8% completeness [13]. Consequently, approximately 1.2% of procedures (both primary and revisions) are not recorded. Of these missing joint replacement procedures, a small proportion 0.03% (i.e., ~2.5% of 1.2%) are expected to be revision procedures rather than primary procedures. This equates to approximately 59 missing revisions procedures (50 XLPE and 9 AOXLPE) from the current data set. These missing procedures are likely to be missing completely at random (MCAR) and given their small proportion and the survival analysis approach used, this is expected to have a negligible impact on the validity or precision of the study’s findings.

As collection of surgical approach information began in 2015, surgical approach for the first year of the study period was missing (for 19,018 procedures). The unadjusted and adjusted analyses were conducted excluding procedures with unknown surgical approach so that these analyses were comparable (i.e., complete case analyses were undertaken).

Comparisons were made between the 2 groups for revisions for loosening, revisions for “wear-related causes” (which included loosening, insert wear, osteolysis, and breakage of acetabular insert), revisions for fracture, revisions for infection, and revisions for dislocation. Sub-analyses were carried out stratifying by age group, femoral head size and type, femoral fixation, and surgical approach. Statistical analysis was performed using SAS software version 9.4 (SAS Institute, Cary, NC, USA).

### Ethics, data sharing plan, funding, use of AI, and disclosures

The AOANJRR is approved by the Commonwealth of Australia as a federal quality assurance activity under section 124X of the Health Insurance Act, 1973. All AOANJRR studies are conducted in accordance with ethical principles of research (the Helsinki Declaration II). Registration was not required. No study funding was received. The AOANJRR is funded by the Australian Federal Government Department of Health and Aged Care. AI tools were not used.

The authors declare no potential conflicts of interest. Complete disclosure of interest forms according to ICMJE are available on the article page, doi: 10.2340/17453674.2025.45181

## Results

There were 469,012 eligible THR with either XLPE or AOXLPE inserts. After exclusions for diagnoses other than osteoarthritis, procedures prior to 2014, or those that used either cemented acetabular components, ceramicized metal heads, non-modular, constrained, or dual mobility designs, and those with an unknown surgical approach, there were 198,073 THR procedures with 35,309 AOXLPE and 162,764 XLPE inserts included in the study (see Figure 1). There were 769 and 4,327 revisions of THR with AOXLPE and XLPE inserts respectively. Demographic and prosthesis factors are summarized in Table 1. While mean ages, sex distribution, and BMI were similar for the 2 groups, there were inequalities in prosthesis factors. The AOXLPE group had a higher proportion with ceramic heads (77% vs 52%), head size > 32 mm (81% vs 49%), and cementless femoral components (81% vs 56%).

**Table 1:** Summary of primary total hip replacement by bearing surface. Values are count (%) or as specified

Variable	XLPE	AOXLPE	Total
Follow-up, years			
Mean (SD)	4 (2.5)	2.9 (2.2)	3.8 (2.5)
Median (IQR)	4 (1.8–6.1)	2.6 (1.1–4.4)	3.6 (1.6–.9)
Minimum	0	0	0
Maximum	9.9	9.2	9.9
Age			
Mean (SD)	69 (10)	68 (10)	69 (10)
Median (IQR)	70 (62–76)	68 (61–75)	70 (62–76)
Age group			
< 55	14,584 (9.0)	3,438 (9.7)	18,022 (9.1)
55–64	36,751 (23)	8,936 (25)	45,687 (23)
65–74	60,402 (37)	13,649 (39)	74,051 (37)
≥ 75	51,027 (31)	9,286 (26)	60,313 (30)
Sex			
Male	76,025 (47)	16,922 (48)	92,947 (47)
Female	86,739 (53)	18,387 (52)	105,126 (53)
Body mass index category **^a^**			
Underweight	1,085 (0.7)	264 (0.8)	1,349 (0.7)
Normal	30,841 (20)	7,525 (22)	38,366 (20)
Pre obese	57,187 (36)	12,904 (37)	70,091 (36)
Obese class 1	41,630 (26)	8,592 (25)	50,222 (26)
Obese class 2	18,056 (12)	3,699 (11)	21,755 (11)
Obese class 3	8,678 (5.5)	1,708 (4.9)	10,386 (5.4)
ASA score **^b^**			
1	10,911 (6.7)	3,153 (8.9)	14,064 (7.1)
2	86,223 (53)	19,386 (55)	105,609 (53)
3	63,108 (39)	12,331 (35)	75,439 (38)
4	2,286 (1.4)	394 (1.1)	2,680 (1.4)
5	12 (0)	1 (0)	13 (0)
Head size			
< 32 mm	7,711 (4.7)	235 (0.7)	7,946 (4.0)
32 mm	72,784 (45)	6,584 (19)	79,368 (40)
> 32 mm	82,269 (51)	28,490 (81)	110,759 (56)
Head material			
Ceramic	88,358 (54)	27,439 (78)	115,797 (59)
Metal	74,406 (46)	7,870 (22)	82,276 (41)
Acetabular fixation			
Cementless	162,764 (100)	35,309 (100)	198,073 (100)
Femoral fixation			
Cementless	91,947 (57)	28,397 (80)	120,344 (61)
Cemented	70,817 (43)	6,912 (20)	77,729 (39)
Surgical approach			
Anterior	34,537 (21)	9,737 (28)	44,274 (22)
Lateral	27,430 (17)	4,192 (12)	31,622 (16)
Posterior	100,797 (62)	21,380 (61)	122,177 (62)
Total	162,764	35,309	198,073

ASA: American Society of Anesthesiologists physical status classification, IQR: interquartile range, SD: standard deviation.

a Excludes 5,904 procedures with unknown body mass index category. Categories are underweight: < 18.5, normal: 18.5– < 25, pre obese: 25– < 30, obese class 1: 30– < 35, obese class 2: 35– < 40, and obese class 3: ≥ 40.

b Excludes 268 procedures with unknown ASA Score.

AOXLPE use increased to 30% of hip procedures in 2023, with shorter median follow-up for this group. The acetabular components which used AOXLPE or XLPE inserts are indicated in Appendix Table 1A.

The reasons for revision for the 2 groups are presented in Table 2 while Table 3 lists the revision procedures.

**Table 2:** Revision diagnosis of primary total hip replacement by bearing surface (primary diagnosis osteoarthritis)

Revision diagnosis	XLPE		AOXLPE	
	Primaries revised n (%)	% of revisions	Primaries revised n (%)	% of revisions
Infection	1,368 (0.8)	32	260 (0.7)	34
Prosthesis dislocation/instability	1,022 (0.6)	24	197 (0.6)	26
Fracture	891 (0.5)	21	148 (0.4)	19
Loosening	641 (0.4)	15	93 (0.3)	12
Pain	73 (0.0)	1.7	11 (0.0)	1.4
Leg length discrepancy	71 (0.0)	1.6	23 (0.1)	3.0
Malposition	64 (0.0)	1.5	13 (0.0)	1.7
Implant breakage stem	25 (0.0)	0.6	2 (0.0)	0.3
Incorrect sizing	25 (0.0)	0.6	5 (0.0)	0.7
Implant breakage acetabular insert	22 (0.0)	0.5	2 (0.0)	0.3
Lysis	16 (0.0)	0.4		
Metal-related pathology	11 (0.0)	0.3		
Heterotopic bone	8 (0.0)	0.2	1 (0.0)	0.1
Implant breakage acetabular	8 (0.0)	0.2		
Wear acetabular insert	6 (0.0)	0.1	1 (0.0)	0.1
Tumor	5 (0.0)	0.1	1 (0.0)	0.1
Implant breakage head	2 (0.0)	0.0		
Progression of disease	1 (0.0)	0.0		
Wear acetabulum	1 (0.0)	0.0		
Wear head	1 (0.0)	0.0		
Other	66 (0.0)	1.5	12 (0.0)	1.6
Revisions	4,327 (2.7)	100	769 (2.2)	100
Primary THRs	162,764		35,309	

**Table 3:** Type of revision of primary total hip replacement by bearing surface (primary diagnosis osteoarthritis)

Type of evision	XLPE		AOXLPE	
	Primaries revised n (%)	% of revisions	Primaries revised n (%)	% of revisions
Head and insert	1,456 (0.9)	34	276 (0.8)	36
Femoral component	1,342 (0.8)	31	231 (0.7)	30
Acetabular component	645 (0.4)	15	116 (0.3)	15
THR (femoral and acetabular)	411 (0.3)	9.5	81 (0.2)	11
Head only	208 (0.1)	4.8	40 (0.1)	5.2
Cement spacer	122 (0.1)	2.8	11 (0.0)	1.4
Minor components	68 (0.0)	1.6	5 (0.0)	0.7
Insert only	48 (0.0)	1.1	7 (0.0)	0.9
Removal of prostheses	18 (0.0)	0.4	1 (0.0)	0.1
Reinsertion of components	7 (0.0)	0.2		
Bipolar head and femoral	2 (0.0)	0.0	1 (0.0)	0.1
Revisions	4,327 (2.7)	100	769 (2.2)	100
Primary THRs	162,764		35,309	

### Outcome

**Figure 2:**
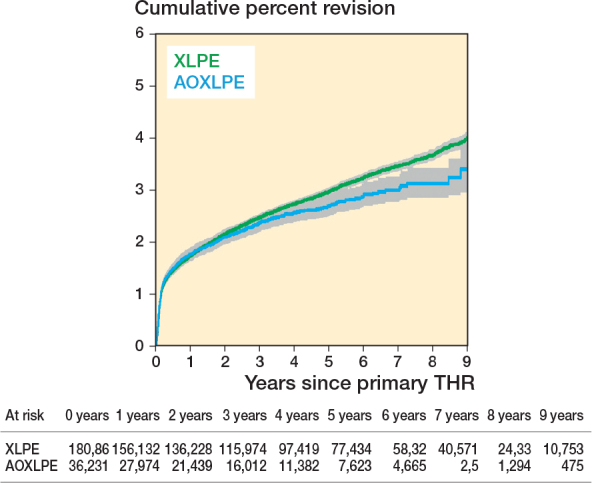
Cumulative percentage revision of primary total hip replacement by bearing surface.

There was no early difference between the 2 groups in the rate of all-cause revision but after 3 years THR using AOXLPE inserts had a lower revision rate than THR using XLPE (Figure 2). The unadjusted and adjusted comparisons (adjusting for age, sex, head size, head material, femoral fixation, and surgical approach) were similar, with the unadjusted HR becoming significant after 3 years (HR 0.64, CI 0.48–0.84) P = 0.002 as did the adjusted HR 0.63 (CI 0.47–0.83) P = 0.001 (Table 4).

**Table 4:** Hazard ratios (HR) of revision primary total hip replacement by bearing surface (primary diagnosis osteoarthritis) with 95% confidence intervals (CI)

AOXLPE vs XLPE Time period	Unadjusted HR (CI)	P value	Adjusted HR (CI) ^a^	P value
0–3 months	1.01 (0.91–1.12)	0.9	0.98 (0.88–1.09)	0.7
3–6 months	0.94 (0.73–1.21)	0.6	0.91 (0.71–1.18)	0.5
6–9 months	1.08 (0.79–1.47)	0.6	1.05 (0.77–1.43)	0.7
9–36 months	0.87 (0.74–1.03)	0.1	0.85 (0.72–1.01)	0.06
>36 months	0.64 (0.48–0.84)	0.002	0.63 (0.47–0.83)	0.001

a Adjusted for age, sex, head size, head material, femoral fixation, and surgical approach.

Further analyses showed that there were lower rates of revision for loosening (after 2 years), wear-related causes (also after 2 years), and fracture for THR with AOXLPE inserts. There were no differences when comparing the groups for revisions for dislocation/instability or revisions for infection (Table 5).

**Table 5:** Summary of revisions of primary total hip replacement by reasons for revision and bearing surface

Reason for revision									Time period months	HR (CI)	P value	Adjusted HR (CI)	P value
Insert material	Revised	Total	1-year CPR (CI)	At risk	5-year CPR (CI)	At risk	8-year CPR (CI)	At risk					
Loosening													
AOXLPE	93	35,309	0.2 (0.2–0.3)	27,108	0.3 (0.3–0.4	6,895	0.4 (0.3–0.5)	686	0–24	1.05 (0.83–1.33)	0.7	0.87 (0.69–1.11)	0.3
XLPE	641	162,764	0.2 (0.2–0.2)	138,540	0.5 (0.4–0.5)	61,601	0.6 (0.6–0.7)	11,121	> 24	0.28 (0.14–0.54)	< 0.001	0.22 (0.11–0.44)	< 0.001
Wear-related causes													
AOXLPE	96	35,309	0.2 (0.2–0.3)	27,108	0.4 (0.3–0.4)	6,895	0.4 (0.3–0.5)	686	0–24	1.03 (0.82–1.30)	0.8	0.86 (0.68–1.09)	0.2
XLPE	685	162,764	0.2 (0.2–0.2)	138,540	0.5 (0.5–0.5)	61,601	0.7 (0.6–0.8)	11,121	> 24	0.26 (0.13–0.50)	< 0.001	0.21 (0.11–0.40)	< 0.001
Fracture													
AOXLPE	148	35,309	0.3 (0.3–0.4)	27,108	0.5 (0.4–0.6)	6,895	0.7 (0.6–1.0)	686	E.P.	0.89 (0.75–1.06)	0.2	0.80 (0.67–0.96)	0.02
XLPE	891	162,764	0.4 (0.3–0.4)	138,540	0.6 (0.6–0.6)	61,601	0.8 (0.8–0.9)	11,121					
Dislocation/Instability													
AOXLPE	197	35,309	0.4 (0.4–0.5)	2,7108	0.7 (0.6–0.8)	6,895	0.8 (0.7–0.9)	686	E.P.	1.00 (0.86–1.17)	1	1.14 (0.97–1.34)	0.1
XLPE	1,022	162,764	0.4 (0.4–0.5)	138,540	0.7 (0.7–0.8)	61,601	0.8 (0.8–0.9)	11,121					
Infection													
AOXLPE	260	35,309	0.7 (0.6–0.8)	27108	0.8 (0.7–1.0)	6,895	0.9 (0.8–1.0)	686	E.P.	0.95 (0.83–1.08)	0.4	0.97 (0.84–1.11)	0.7
XLPE	1,368	162,764	0.7 (0.6–0.7)	138540	0.9 (0.9–1.0)	61,601	1.0 (1.0–1.1)	11,121					

Note: Cumulative percentage revision (CPR) displayed with 95% confidence intervals (CI). Hazard ratios (HR = AOXLPE:XLPE) presented both unadjusted and adjusted for age, sex, femoral fixation, femoral head size and material, and surgical approach with CIs. E.P.: entire period.

In sub-analyses that compared AOXLPE with XLPE inserts by prosthesis factors, a higher revision rate was found for the AOXLPE group with femoral head sizes ≤ 32 mm, while a lower revision rate was seen with AOXLPE for head size > 32mm after 1.5 years. Procedures using ceramic femoral heads had a lower revision rate with AOXLPE than with XLPE inserts, while metal femoral heads used with AOXLPE inserts had a higher revision rate until 3 months when compared with XLPE. Cemented femoral fixation was associated with a higher revision rate with AOXLPE inserts, while cementless femoral fixation had a lower revision rate with AOXLPE inserts. The anterior surgical approach had a lower revision rate using AOXLPE inserts, but there were no between-group differences with the lateral or posterior approaches (Table 6).

**Table 6:** Summary of revisions of primary total hip replacement by prosthesis factors and bearing surface

Prosthesis factor									Time period months	HR (CI)	P value	Adjusted HR (CI)	P value
Insert material	Revised	Total	1-year CPR (CI)	At risk	5-year CPR (CI)	At risk	8-year CPR (CI)	At risk					
Head size < 32 mm													
AOXLPE	10	235	3.5 (1.7–6.8)	171		10		2	E.P.	1.11 (1.04–1.18)	0.001	1.11 (1.04–1.18)	0.002
XLPE	271	7,711	2.3 (2.0–2.6)	6,876	3.7 ( 3.3–4.2)	3,626	4.3 (3.8–4.9)	858					
Head size 32 mm													
AOXLPE	158	6,584	1.8 (1.5–2.2)	5,224	3.0 (2.5–3.5)	1,271	3.3 (2.7–4.1)	131	E.P.	1.61 (0.86–3.03)	0.1	1.91 (1.01–3.60)	0.04
XLPE	1,893	72,784	1.6 (1.5–1.7)	64,515	2.8 (2.7–2.9)	32,297	3.4 (3.2–3.6)	6,018					
Head size > 32 mm													
AOXLPE	601	28,490	1.7 (1.5–1.9)	21,713	2.6 (2 .4–2.8)	5,614	3.0 (2.7–3.3)	553	0–3	0.96 (0.85–1.09)	0.5	0.95 (0.84–1.08)	0.4
XLPE	2,163	82,269	1.8 (1.7–1.9)	67,149	3.0 (2.9–3.2)	25,678	3.9 (3.7–4.1)	4,245	3–18 > 18	0.92 (0.77–1.09) 0.65 (0.53–0.80)	0.3 < 0.001	0.91 (0.76–1.08) 0.65 (0.53–0.80)	0.3 < 0.001
Ceramic head													
AOXLPE	540	27,439	1.5 (1.4–1.7)	21,124	2.4 (2.2–2.7)	5,517	2.8 (2.5–3.2)	569	E.P.	0.86 (0.78–0.94)	0.001	0.82 (0.75–0.91)	<0.001
XLPE	2,231	88,358	1.7 (1.6–1.8)	73,293	2.9 (2.8–3.0)	29,123	3.5 (3.4–3.7)	4,678					
Metal head													
AOXLPE	229	7,870	2.4 (2.1–2.8)	5,984	3.5 (3.1–4.0)	1,378	3.8 3.2–4.5)	117	0–3	1.48 (1.24,1.76)	< 0.001	1.39 (1.16–1.66)	< 0.001
XLPE	2,096	74,406	1.8 (1.7–1.9)	65,247	3.0 (2.9–3.1)	32,478	3.8 (3.6–4.0)	6,443	> 3	0.98 (0.79,1.22)	0.9	0.93 (0.74–1.15)	0.5
Femur cementless													
AOXLPE	598	28,397	1.7 (1.6–1.9)	21810	2.5 (2.3–2.8)	5,810	2.9 (2.6–3.2)	607	E.P.	0.82 (0.75–0.90)	< 0.001	0.84 (0.76–0.92)	< 0.001
XLPE	2,690	91,947	1.9 (1.8–2.0)	78,434	3.2 (3.1,–3.4)	35,431	3.9 (3.7–4.1)	6,242					
Femur cemented													
AOXLPE	171	6,912	1.9 (1.6–2.2)	5,298	3.3 (2.8–3.9)	1,085	4.0 (3.0–5.2)	79	E.P.	1.23 (1.05–1.44)	0.01	1.26 (1.08–1.48)	0.004
XLPE	1,637	70,817	1.5 (1.4–1.6)	60,106	2.6 (2.5,–2.7)	34,622	3,4 (3.2–3.6)	4,879					
Anterior approach													
AOXLPE	161	9,737	1.3 (1.1–1.6)	7,696	1.9 (1.6–2.3)	2,268	2.4 (1.9–3.1)	220	E.P.	0.70 (0.60–0.83)	< 0.001	0.68 (0.58–0.81)	< 0.001
XLPE	853	34,537	1.6 (1.5–1.8)	28,039	2. 9(2.7–3.1)	10,514	3.8 (3.5–4.2)	1,658					
Lateral approach													
AOXLPE	94	4,192	1.7 (1.3–2.2)	3,377	2.6 (2.1–3.2)	1,090	3.0 (2.3–3.9)	139	E.P.	0.85 (0.69–.05)	0.1	0.84 (0.68–1.04)	0.1
XLPE	839	27,430	1.9 (1.7–2.0)	24,551	3,2 (3.0–3.4)	13,665	3.8 (3.5–4.1)	3,001					
Posterior approach													
AOXLPE	514	21,380	1.9 (1.7–2.1)	16035	3.1 (2.8–3.4)	3537	3.3 ( 3.0–3.7)	327	E.P.	1.07 (0.97–1.18)	0.2	1.03 (0.94–1.14)	0.5
XLPE	2,635	100,797	1.7 (1.7–1.8)	85,950	2.9 (2.8–3.0)	37,422	3.6 (3.5–3.8)	6,462					

Note: See Table 5 and CPR not displayed if < 45 procedures at risk.

### Sensitivity analysis

As data for surgical approach was incomplete, a sensitivity analysis was conducted in which those procedures with unknown surgical approaches formed another categorical level. The results of these analyses were similar to the analyses that excluded those with unknown surgical approach (Appendix Table 2A). There were no material changes in significance or HR that would affect the interpretation of the results.

## Discussion

We aimed to compare the revision rates of THR using cementless acetabular components where the insert was made of either XLPE or AOXLPE, using data from AOANJRR.

We found that THR using AOXLPE acetabular inserts had a lower all-cause revision rate than XLPE inserts after 3 years. This was consistent for both the crude and multiply adjusted comparisons. Not only does AOXLPE appear to be safe to use, but this finding gives indirect support to the concept that antioxidant may improve the wear-resistance of XLPE.

Revisions for loosening, wear-related causes, and fracture were lower with AOXLPE inserts. While early loosening may be due to a failure to gain initial fixation, revisions for late loosening, as well as wear-related causes and fracture, may be consequences of polyethylene wear and osteolysis. In addition, procedures using larger diameter and ceramic femoral heads, cementless femoral fixation, and the anterior surgical approach showed lower revision rates with AOXLPE. These prosthesis and technique features are commonly selected for higher demand patients, where wear-related performance differences can be more readily detected.

While we found differences in revision rates, with many favoring AOXLPE, these findings were not universal, and the magnitude of difference was often small. Additionally, the analyses of cemented femoral stems and procedures using metal femoral heads showed lower revision rates with XLPE inserts. Selection of lower cost options (such as XLPE, cemented femoral fixation, and metal heads) are more common for older, less active patients, where revisions are more likely due to non-wear-related reasons. This indicates the differences found in overall revision may not be totally due to the insert but, in part, to the femoral or acetabular components used as well.

Previous comparisons of AOXLPE and XLPE have been conflicting, with some revealing promising in vivo wear results for AOXLPE [6,14] measured by femoral head penetration [8,15], while others have shown no difference in overall migration [7] or migration after the bedding-in period [16]. Our results contrast with previous clinical trials. A multicenter study comparing 520 hips with AOXLPE inserts with 457 hips with XLPE showed similar revision rates at 7 years [9], while another comparing AOXLPE monoblock cups with a similar cup of UHMWPE found a 98% 6-year survival for both groups [15]. These studies were designed to compare polyethylene wear, but both were underpowered regarding revision rates. Two meta-analyses that reported THR revision results revealed no difference between AOXLPE and XLPE [17,18], while another showed AOXLPE liners lowered the revision risk at 5–7 years, but this finding was disproportionately influenced by a single study [12].

Registry studies have discerned a difference in revision rate when comparing XLPE with UHMWPE [2,19,20], but have provided limited evidence regarding AOXLPE.

Our findings differ from a Finnish registry study, which found 94% survival of hips using AOXLPE at 7 years, while their moderately cross-linked comparison group was comparable at 93% [21]. Their 2 groups also had similar survivorship when analyzed for wear-related reasons for revision. Kjaergaard et al. compared polyethylene inserts in Demark, finding fewer polyethylene-related revisions for THR using AOXLPE but a higher all-cause revision rate for the first 3 months, largely for femoral loosening and periprosthetic fracture and attributed this to the femoral components used [22]. Compared with our study, these studies had shorter follow-up, smaller study populations and were both restricted to products from a single manufacturer with limited choices of femoral and acetabular components. While restricting inserts may have been intended to limit confounding, it reduces the ability to generalize findings for entire groups.

### Strengths

The strengths of our study are the large patient cohort, inclusion of all variants of the 2 polyethylene types, used with contemporary components, with medium-term follow-up of the protheses of interest, and analyses adjusting for possible confounding factors, when used in a community setting.

### Limitations

We have used all-cause revision as the outcome measure, and there are some reasons for revision that are unrelated to the type of polyethylene used. Sub-analyses were consequently performed selecting specific reasons for revision that have a closer relationship to polyethylene wear. Unfortunately, we had too few procedures for prosthesis-specific analyses. Additionally, we have grouped the acetabular inserts into 2 polyethylene categories, and there may be performance differences within each group. Furthermore, the median follow-up duration for the AOXLPE group (2.6 years) was shorter than for the XLPE group (4.3 years) and it is likely that wear-related differences may become more evident with longer follow-up.

### Conclusion

While there was no early difference, THR with AOXLPE acetabular inserts had a lower revision rate after 3 years than THR with XLPE. This suggests a possible clinical benefit using AOXLPE but the difference may, in part, be related to the associated femoral or acetabular components.

### Supplementary data

Supplementary Tables 1–2 and a DAG are available on the article page, doi: 10.2340/17453674.2025.45181

## Supplementary Material



## Notes

PLL: Conceptualization, study design, data acquisition, data interpretation, manuscript preparation. DGC, PNS, RDS: Data interpretation, manuscript editing. HO, PD: Data analysis, manuscript editing.

The authors would like to acknowledge the AOANJRR for provision of the data used in this study, the surgeons and patients who allowed data collection, and Sophie Corfield for her assistance with manuscript preparation.

Handling co-editors: Keijo Mäkelä and Robin Christensen

*Acta* thanks Matias Mikael Hemmilä and Karin Svensson Malchau for help with peer review of this manuscript.
