# Supplementary material for: Association of antioxidant-added highly cross-linked polyethylene on revision risk: a registry-based study of 198,073 total hip replacements from the Australian Orthopaedic Association National Joint Replacement Registry between 2014 and 2023
Source: Acta Orthop. 2026 Jan 23;97:28–34. doi: 10.2340/17453674.2025.45181 (PMC12829337; doi:10.2340/17453674.2025.45181)
Supplement: Supplementary file 1 [file ActaO-97-45181-s1.pdf]

Supplementary Data

Supplementary Table 1. Revised number of primary total hip replacement by prosthesis model and bearing surface

Acetabular model	Acetabular manufacturer	Bearing surface	Inset model	Revised	Total
Acetabular Shell (Global)	Global Orthopedic Technology	XLPE	Acetabular liner (Global)	90	3,223
Allofit	Zimmer Biomet	XLPE	Durasul	85	2,576
Allofit	Zimmer Biomet	XLPE	Longevity	1	158
Allofit	Zimmer Biomet	AOXLPE	Vivacit-E	0	132
Atlas (Shell)	Fournitures Hospitalières Groupe	XLPE	Atlas	2	51
Austral	Amplitude	XLPE	Austral	20	624
Continuum	Zimmer Biomet	XLPE	Longevity	200	4,843
Continuum	Zimmer Biomet	AOXLPE	Vivacit-E	27	1,003
Delta-One-TT	Lima LTO	XLPE	X-Lima Delta	2	30
Delta-TT	Lima LTO	XLPE	X-Lima Delta	30	996
Dynasty	MicroPort	XLPE	A-Class	96	1,922
EP-Fit Plus	Smith & Nephew	XLPE	EPF-Plus	0	23
EP-Fit Plus	Smith & Nephew	XLPE	Rexpol	1	43
Fin II	Gruppo Bioimpianti	XLPE	Fin II	5	185
Fitmore	Zimmer Biomet	XLPE	Durasul	81	2,108
Fixa	Adler Ortho	XLPE	Fixa	29	555
G7	Zimmer Biomet	XLPE	G7 Arcom	1	39
G7	Zimmer Biomet	AOXLPE	G7 E1	263	11,449
Logical G	Signature Orthopaedics	XLPE	Logical	252	7,193
Mpact	Medacta	XLPE	Highcross	337	13,778
PINNACLE	Depuy	XLPE	ALTRX	349	17,937
PINNACLE	Depuy	XLPE	Marathon	916	26,645
Plasmafit	Aesculap	AOXLPE	Vitelene	5	102
Procotyl L	MicroPort	XLPE	Rim-Lock A-Class	28	2,114
R3	Smith & Nephew	XLPE	R3 XLPE	258	7,853
Reflection (Shell)	Smith & Nephew	XLPE	Reflection XLPE	13	693
Trabecular Metal (Shell)	Zimmer	XLPE	Longevity	68	1,804
Trident (Shell)	Stryker Orthopaedics	XLPE	Trident X3	1511	65,361
Trident II (Shell)	Stryker Orthopaedics	XLPE	Trident X3	6	738
Trident II/Tritanium (Shell)	Stryker Orthopaedics	XLPE	Trident X3	43	2,764

Acetabular model	Acetabular manufacturer	Bearing surface	Inset model	Revised	Total
Trident/Tritanium (Shell)	Stryker Orthopaedics	XLPE	Trident X3	209	6,540
Trilogy	Zimmer Biomet	XLPE	Longevity	170	3,494
Trilogy IT	Zimmer Biomet	XLPE	Longevity	14	336
Trilogy IT	Zimmer Biomet	AOXLPE	Vivacit-E	6	152
Trinity	Corin	XLPE	HXLPE	30	1,453
Trinity	Corin	AOXLPE	ECIMA	494	23,182
Trinity Plus	Corin	AOXLPE	ECIMA	0	60
Versafitcup CC	Medacta	XLPE	Highcross	182	4,781
seleXys	Mathys	AOXLPE	Vitamys	9	151
Total		.		5,833	217,091

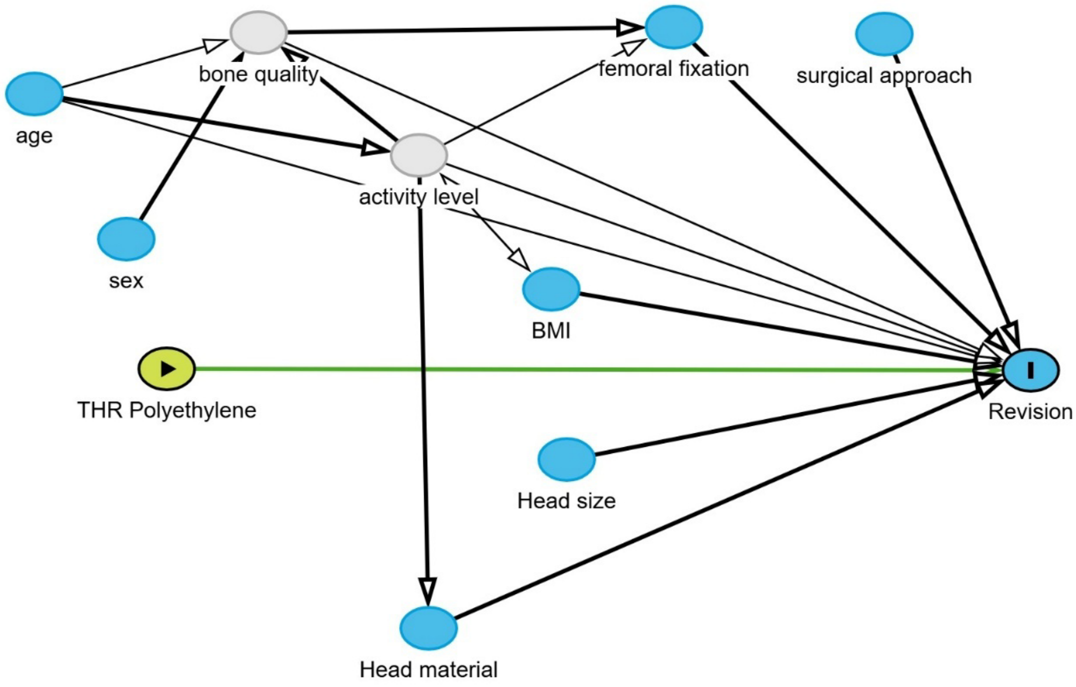

Supplementary Figure. DAG showing relationship of variables to outcome.

Supplementary Table 2. Sensitivity analysis where unknown surgical approach was categorized as "other." Unadjusted and adjusted hazard ratios (HR = AOXLPE: XLPE) of revision in primary total hip replacement by bearing surface (primary diagnosis osteoarthritis)

Time period, months	Unadjusted HR (CI)	P value	Adjusted HR (CI) [a]	P value
0–3	1.03 (0.93–1.13)	0.6	0.93 (0.72–1.19)	0.6
3–6	0.95 (0.74–1.22)	0.7	1.05 (0.78–1.42)	0.7
6–9	1.08 (0.80–1.45)	0.6	0.77 (0.67–0.88)	< 0.001
> 9	0.79 (0.69–0.90)	< 0.001	1.00 (0.90–1.11)	0.9

[a] Adjusted for age, sex, head size, head material, femoral fixation, and surgical approach.
